# Increased local connectivity of brain functional networks during facial processing in schizophrenia: evidence from EEG data

**DOI:** 10.18632/oncotarget.20598

**Published:** 2017-09-01

**Authors:** Tianyi Yan, Wenhui Wang, Tiantian Liu, Duanduan Chen, Changming Wang, Yulong Li, Xudong Ma, Xiaoying Tang, Jinglong Wu, Yiming Deng, Lun Zhao

**Affiliations:** ^1^ School of Life Science, Beijing Institute of Technology, Beijing, China; ^2^ Beijing Advanced Innovation Center for Intelligent Robots and Systems, Beijing Institute of Technology, Beijing, China; ^3^ Beijing Key Laboratory of Mental Disorders, Beijing Anding Hospital, Capital Medical University, Beijing, China; ^4^ Beijing National Day School, Beijing, China; ^5^ Guang Zhou Clifford School, Guang Dong, China; ^6^ Department of Neurology, Beijing Tiantan Hospital, Capital Medical University, Beijing Key Laboratory of Translational Medicine for Cerebrovascular Disease, Beijing, China; ^7^ China National Clinical Research Center for Neurological Diseases, Beijing, China; ^8^ School of Education, Beijing Normal University Zhuhai, Zhuhai, China

**Keywords:** schizophrenia, facial processing, dynamic brain network, phase synchrony, graph theory

## Abstract

Schizophrenia is often considered to be a disconnection syndrome. The abnormal interactions between large-scale functional brain networks result in cognitive and perceptual deficits. The present study investigated event-related functional connectivity networks to compare facial processing in individuals with and without schizophrenia. Faces and tables were presented to participants, and event-related phase synchrony, represented by the phase lag index (PLI), was calculated. In addition, cortical oscillatory dynamics may be useful for understanding the neural mechanisms underlying the disparate cognitive and functional impairments in schizophrenic patients. Therefore, the dynamic graph theoretical networks related to facial processing were compared between individuals with and without schizophrenia. Our results showed that event-related phase synchrony was significantly reduced in distributed networks, and normalized clustering coefficients were significantly increased in schizophrenic patients relative to those of the controls. The present data suggest that schizophrenic patients have specific alterations, indicated by increased local connectivity in gamma oscillations during facial processing.

## INTRODUCTION

Schizophrenia is a mental disorder often characterized by abnormal social behavior, such as difficulty understanding and dealing with social interactions. Social cognition has been globally defined as the ability to perceive the intention and disposition of others and then guide social interaction. During the interactions, facial information plays a key role.

Several lines of research have provided evidence for impaired facial processing in schizophrenia. For example, in terms of the underlying neural correlates of facial processing in schizophrenia, abnormities in the frontal and temporal lobes seem to prevail [1], including reduced activation and volume of the fusiform facial area [2, 3]. In response to neutral faces, schizophrenia patients also show hyper-activations in the frontal and cingulate areas and in the basal ganglia [4]. In a recent meta-analysis of functional neuroimaging data, Li et al. concluded that relative to healthy controls, individuals with schizophrenia show less activation in the bilateral amygdala, the para hippocampal gyrus and fusiform gyrus, the right superior frontal gyrus and the lenti-form nucleus. Schizophrenia patients show more activation in the left insula while performing facial processing tasks [5]. In addition, event-related potential results show that the face-specific N170 is decreased in schizophrenia patients [6–10].

Importantly, synchronous oscillatory neural activity is a possible candidate mechanism for the coordination of neural activity between and within functionally specialized brain regions [11–13]. It has been shown that the coordination of distributed neural activity is dysfunctional in people with schizophrenia [14–17]. For example, Uhlhaas et al. reported that deficits in Gestalt perception in schizophrenia patients are associated with reduced phase synchrony of the beta-band in the 200–300 ms interval involving fronto-temporal and parieto-occipital electrodes, indicating impairment of the long-range synchronization of neural responses, which may reflect a core deficit in the coordination of neural activity and underlie the specific cognitive dysfunctions associated with the disorder [17]. In addition, there is evidence that gamma-band phase synchronization related to higher cognitive processes, such as facial perception, is significantly lower at 200–300 ms in schizophrenia patients [16].

The conventional approach for investigating the functional connectivity graph of the brain is extracting the characteristics of a so-called small world network, a network with a high clustering coefficient (a measure of large-scale network segregation) and low path length (a measure of large-scale network integration) simultaneously [18]. Importantly, recent studies using functional magnetic resonance imaging (fMRI) and electroencephalography (EEG) showed decreased local specialization in schizophrenia, measured as either local efficiency or as the conceptually similar clustering coefficient [14, 19, 20]. Anatomical networks constructed with diffusion tensor tractography (DTI) also found decreases in local efficiency and global efficiency [21, 22]. In structural networks, local and global efficiencies negatively correlate with scores on the Positive and Negative Symptom Scale [21]. The above neuroimaging findings suggest that schizophrenia is indeed associated with a subtle randomization of connection patterns within the brain [23].

It is highly likely that the topological configuration of functional brain networks is dynamically reorganized in the context of changing environmental conditions or different experimental task demands. Previous research has averaged functional connectivity over longer time periods, neglecting the physiological reality that the brain’s information processing system must be rapidly reconfigurable [24]. However, thus far, few studies have directly investigated the dynamics of the topology of the human brain network, and this has remained a methodologically challenging area [25, 26]. Spatially precise hemodynamic neuroimaging measurements, such as fMRI, do not measure neuronal dynamics directly and do not have subsecond time resolution. These technical limitations clearly constrain what fMRIs can reveal about network dynamics on faster time scales, which are important for immediate perception, rapid action, and cognition. EEG analysis is an important method that permits the analysis of coordinating interactions over large distances in the brain with millisecond resolution, which is necessary because synchronization of oscillatory activity in the EEG frequency bands occurs with precision in the millisecond range [12]. As one of the synchronization measurements, the PLI refers to a period of phase locking between two events and can only be estimated in a statistical sense, reflecting the extent of phase variability for a given frequency over time. Actually, the PLI removes and attenuates synchronization that occurs at or near the zero phase difference and thereby reduces the impact of spurious synchronization originating from common sources or volume conduction [27–29]. The present study combined the phase synchrony of electrode interactions and the graph theoretical metrics of network topography to investigate event-related functional connectivity derived from EEG data during facial perception in individuals with and without schizophrenia. The PLI, a linear and nonlinear estimator computed for each pair of sensors, was used to construct graphs. PLI-weighted connectivity networks were calculated according to graph theory and characterized by a normalized clustering coefficient. We hypothesized that reduced event-related phase synchrony and increased event-related clustering coefficients in networks related to facial processing would be observed in schizophrenia patients relative to those of healthy controls.

## RESULTS

**Figure 1:**
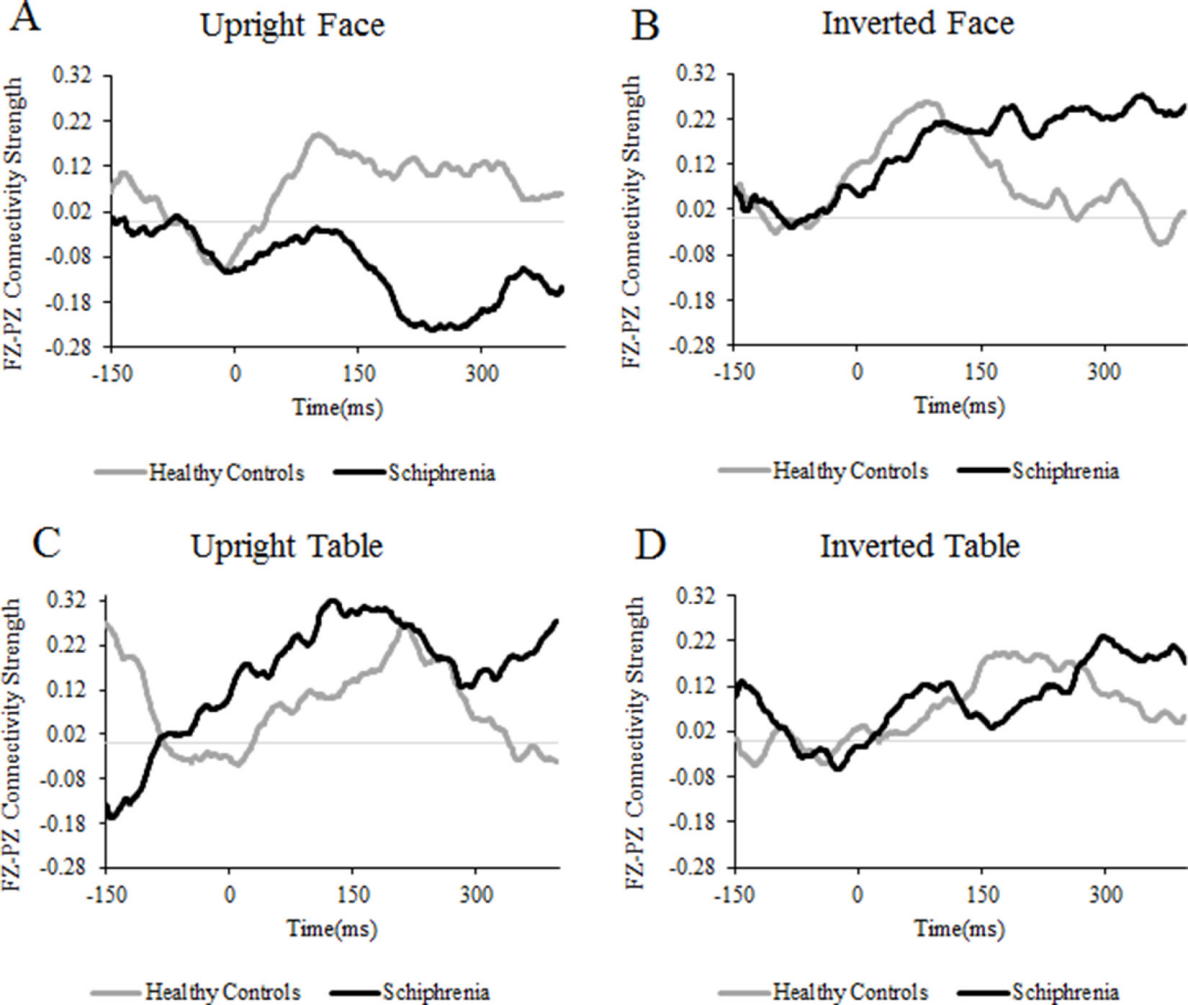
Time courses of connectivity strength Time courses of the gamma-band connectivity strength calculated by the PLI between the Fz and Pz electrode sites averaged across subjects. Values are standard deviations from the 150 ms baseline.

The main results of phase synchrony are summarized in Figure 1. Event-related changes in connectivity were observed in the alpha (8–14 Hz) and beta (15–30 Hz) frequency ranges; however, we did not investigate the alpha and beta activities because there were no significant group effects. For the gamma band, there were clear differences in event-related connectivity between the two groups, especially in the 150–300 ms time window after stimuli onset.

### Topographical analysis of phase synchrony

**Figure 2:**
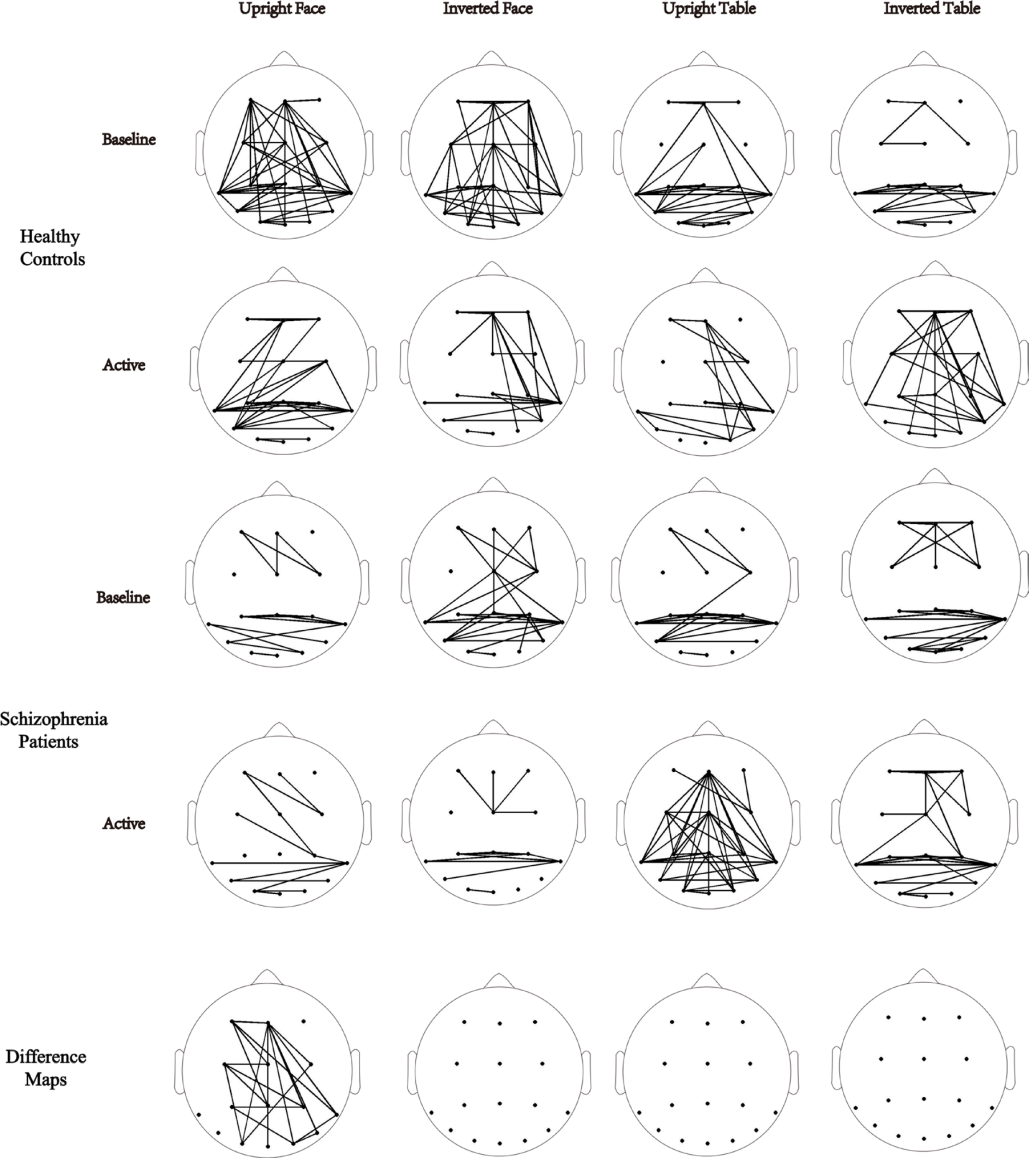
Topography Topography of phase synchrony for all conditions, baseline and time window, respectively, in the 150–300 ms gamma band. Top row, healthy controls. Middle row, patients with schizophrenia. Bottom row, difference map. In the map of the healthy controls and the map of the schizophrenia patients, synchrony between electrodes is indicated by lines, which were drawn only if the synchrony value was beyond the distribution of shuffled data sets (*p* < 0.05). In the difference maps, functional connectivity differences between the patients and the controls involve a decreased distributed network of connections (black edges) for the frontal, central parietal, temporal and occipital areas.

The network-based statistic (NBS) was employed to investigate group differences in event-related phase synchrony during each condition in each active time window for the gamma band. NBS connectivity analysis showed that there were no significant differences between the groups in the 0–150 ms time window. In the 150–300 ms time window for the upright face condition, NBS revealed reduced event-related connectivity in individuals with schizophrenia in a distributed network of brain regions, which included strong involvement of the frontal, central, parietal and occipital brain electrodes (*p <* 0.05, Figure 2). No significant group differences were observed for other stimuli conditions (ps > 0.1). Comparison of connectivity between the baseline and active windows within the schizophrenia and control groups using NBS (ps > 0.1).

### Event-related small-world properties

**Figure 3:**
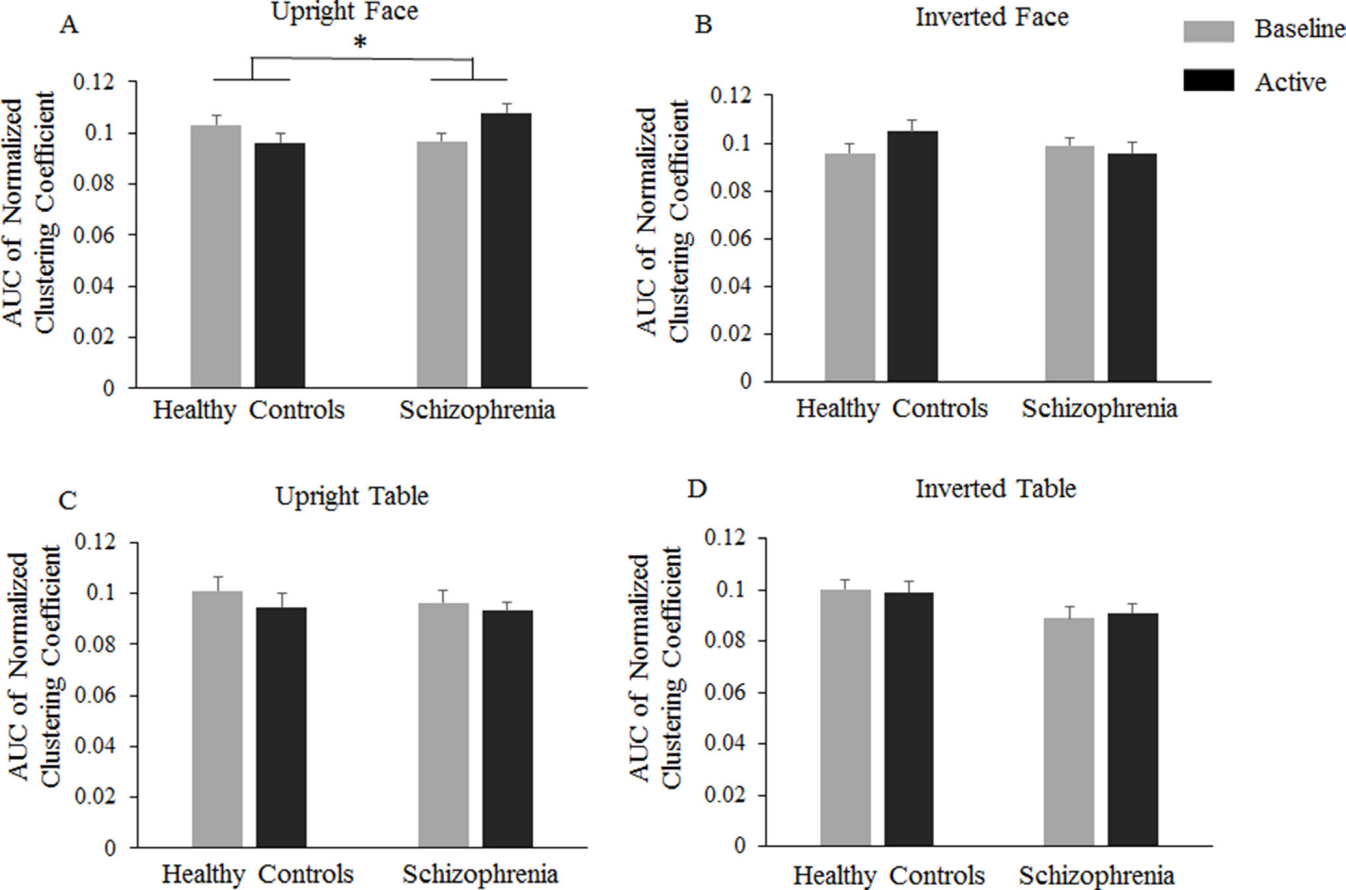
Normalized clustering coefficient differences Bar graphs display the mean (standard error) Event -related normalized clustering coefficients in healthy controls and schizophrenia patients in the –150∼0 ms baseline time window and the 150∼300 ms active time window.

Analysis of the normalized clustering coefficient index (*γ*) revealed a significantly increased coefficient for the schizophrenia group compared to that of the healthy controls for the upright face condition (Figure 3). In the 150–300 ms time window, there were no significant main effects (Group: F(1,47) = 0.456, *P =* 0.503, = 0.010; Orientation: F(1,47) = 0.866, *P =* 0.357, = 0.018; Category: F(1,47) = 0.992, *P =* 0.324, = 0.021). Interestingly, there was a significant OrientationGroup interaction, as F(1,47) = 4.839, *P =* 0.033, and = 0.093, revealing that the event-related clustering coefficient was lower for the upright condition (–0.007) than the inverted condition (0.004; F(1,47) = 5.001, *P =* 0.030, = 0.096) in healthy controls, whereas stimuli orientation did not modulate the event-related clustering coefficient in the schizophrenia group (0.004 and -0.001 for the upright and inverted conditions, respectively; F(1,47) = 0.789, *P =* 0.379, = 0.017). The three-way interaction of OrientationCategoryGroup was also significant, as F(1,47) = 4.184, *P =* 0.046, and = 0.082. Further analysis showed that the OrientationGroup interaction was significant for face stimuli (F(1,47) = 11.368, *P =* 0.002, = 0.195) but not for table stimuli (F(1,47) < 1). In the controls, the event-related clustering coefficient was significantly lower for the upright faces (–0.0069) than for the inverted faces (0.0091; *P =* 0.025), whereas in schizophrenia patients, the event-related clustering coefficient was significantly higher for upright faces (0.0111) than for inverted faces (–0.0030; *P =* 0.024). For upright conditions, the event-related clustering coefficient was significantly lower in healthy controls (–0.0069) than in schizophrenia patients (0.0111; *P =* 0.017), and for inverted conditions, there was no significant group difference (0.0091 and –0.003 for healthy controls and schizophrenia patients, respectively; *P =* 0.119).

## DISCUSSION

The goal of the present study was to clarify the characteristics of facial processing in schizophrenia patients by analyzing phase synchronization and the graph theoretical network of the gamma band. The results of this study support the hypothesis that patients with schizophrenia are characterized by dysfunctions in the long-range coordination of neural activity and local connectivity of the graph theoretical network, as indicated by significant reductions in event-related phase synchrony and increases in event-related normalized clustering coefficients during facial processing.

The present findings of phase synchrony are consistent with previous research showing that long-range coordination occurs in the gamma-band range [10, 30–35]. Gamma -band oscillations have been implicated repeatedly in a wide variety of cognitive and sensory processes, including feature binding and stimulus identification processes, which indicates fast reorganization in response to visual signals [13]. The preferred time window of 150 ms, which we choose to resolve network changes, was too short of a time frame to allow the dynamic network changes in the lower frequency bands to be investigated. Importantly, we found that phase synchrony was distinguished between the schizophrenia patients and the healthy controls during upright face perception, which is in agreement with previous reports [16, 17]. Synchrony patterns in the gamma-frequency range in patients with schizophrenia were mainly reduced in the 150–300 ms interval relative to that of the controls, suggesting that patients with schizophrenia fail to coordinate neural activity among the central, frontal, parietal, and occipital brain regions to produce a fully integrated perception. Actually, there is evidence that the effective communication structure is mechanistically implemented by the pattern of phase-locking among oscillations in communicating neuronal groups [11]. The present patients with schizophrenia demonstrated a selective deficit in facial perception, as a reduction in phase synchrony was observed, suggesting that dysfunction in phase synchrony has a causal role in the cognitive deficits in schizophrenia. Generally, synchrony measures the relation between the temporal signal structures regardless of signal amplitude, and two signals are said to be synchronous if their rhythms coincide. In fact, patients with schizophrenia are characterized by their tendency to exhibit desynchronized neural activity, suggesting a marked dysfunction not only in the initiation but also in the maintenance of synchronous oscillatory neural activity [17].

In addition, the present study shows that on average, the PLI-weighted functional networks exhibited increased clustering coefficients in people with schizophrenia relative to those of healthy controls. The increased clustering coefficients in schizophrenia were associated with reduced long-range phase synchrony, implying relatively stronger local connectivity in schizophrenia. Higher local connectivity is correlated with more regions of the brain neuronal network that need to be isolated from the others, and coincidentally, these regions need to have a dense synchronization among their own sub-parts. Neural networks in schizophrenia patients had less connections between clusters and more connections within clusters to achieve a level of network integration equivalent to that of controls. The presence of clusters in functional networks suggests an organization of statistical dependency indicative of segregated neural processing [36]. This study reports a network topology in highly segregated but poorly integrated networks, revealing abnormalities in the large-scale topology of functional connectivity networks.

Graph theory is a useful tool for its simplicity and generalizability. For example, the hubs of macroscopic brain structural networks derived from tract-tracing data show distinct microscopic properties related to neuronal morphology and density. Associations have been found between network topology and local gene expression profiles, and the hubs of human functional MRI networks have been located in brain regions that have high rates of glucose metabolism, as determined by positron emission tomography (PET). In the present study, the increased normalized clustering coefficient in schizophrenia patients while processing faces may be a good guide for micro research.

The current data also allow for the primary conclusion of the relationship between deficits in large-scale integration and the symptoms of schizophrenia. Additional studies are needed that link the deficits in large-scale network integration to anatomical and neurophysiological dysfunctions, as well as to clinical parameters that have been associated with coordination failure in schizophrenia, which may potentially lead to a new therapeutic perspective on the disorder [17].

Based on the present experimental design, it is possible to elucidate whether the observed impairment is a face-specific phenomenon. The data showed increased event-related local connectivity in schizophrenia patients relative to that of healthy controls for only the upright face condition. Specifically, in healthy controls, the event-related local connectivity for the inverted face condition was significantly increased relative to that of the upright face condition, whereas in schizophrenia patients, the event-related local connectivity in the inverted face condition was significantly reduced relative to that of the upright face condition. The data indicate that lower local connectivity related to configurable facial processing reflected by facial inversion tends to promote long-range connectivity in healthy controls, and higher local connectivity tends to impair long-range connectivity in schizophrenia patients. In line with the phase synchrony results, there was a significantly reduced long-range connectivity in patients with schizophrenia. To the best of our knowledge, this is the first study to show increased event-related local connectivity in networks constructed by the graph theory in schizophrenia patients when viewing pictures of real human faces.

Although group differences in the gamma frequency range synchronization were evident during the perception of the upright face, the approach of using NBS for characterizing network connectivity differences did not support the direct analysis of interactions [28]. Furthermore, it is only valid to make inferences about the component as a whole [37, 38] as a consequence of weak control of the familywise error rate (FWER) [37, 38]. Thus, while the NBS can offer a considerable gain in statistical power, this gain comes at the expense of not being able to localize the effects to individual edges [36, 38]. Accordingly, it is possible that patients with schizophrenia may express different network connectivities during the processing of inverted faces, upright tables and inverted tables relative to those of the controls. Additional studies are needed that link deficits in phase synchrony to anatomical and neurophysiological dysfunction as well as to clinical parameters that have been associated with coordination failure in schizophrenia.

## MATERIALS AND METHODS

### Participants

Twenty-five patients with schizophrenia (10 females; mean 31.1 ± 10.8 y) and 25 age-matched healthy controls (10 females; mean 31.9 ± 10.5 y) participated in this study. Each patient was diagnosed with schizophrenia according to the DSM-IV (Diagnostic and Statistical Manual of Mental Disorders, Fourth Edition). None of the included patients had a history of severe medical disorders or severe neurological disorders. A trained psychiatrist or psychologist evaluated the psychiatric symptoms on the Positive and Negative Syndrome Scale (PANSS). The Personal and Social Performance (PSP) scale was used to assess the social functioning ability of each of the participants, which was also found to be an acceptable, quick and valid measure of their personal and social functioning ability.

The healthy volunteers had no history of any major psychiatric disorders or major physical illnesses and were not taking any medications that affect the central nervous system. This study was approved by the Institutional Review Board of the Beijing Institute of Technology. All participants received payment for their participation and gave their informed consent prior to the experiment.

### Stimuli and procedure

**Figure 4:**
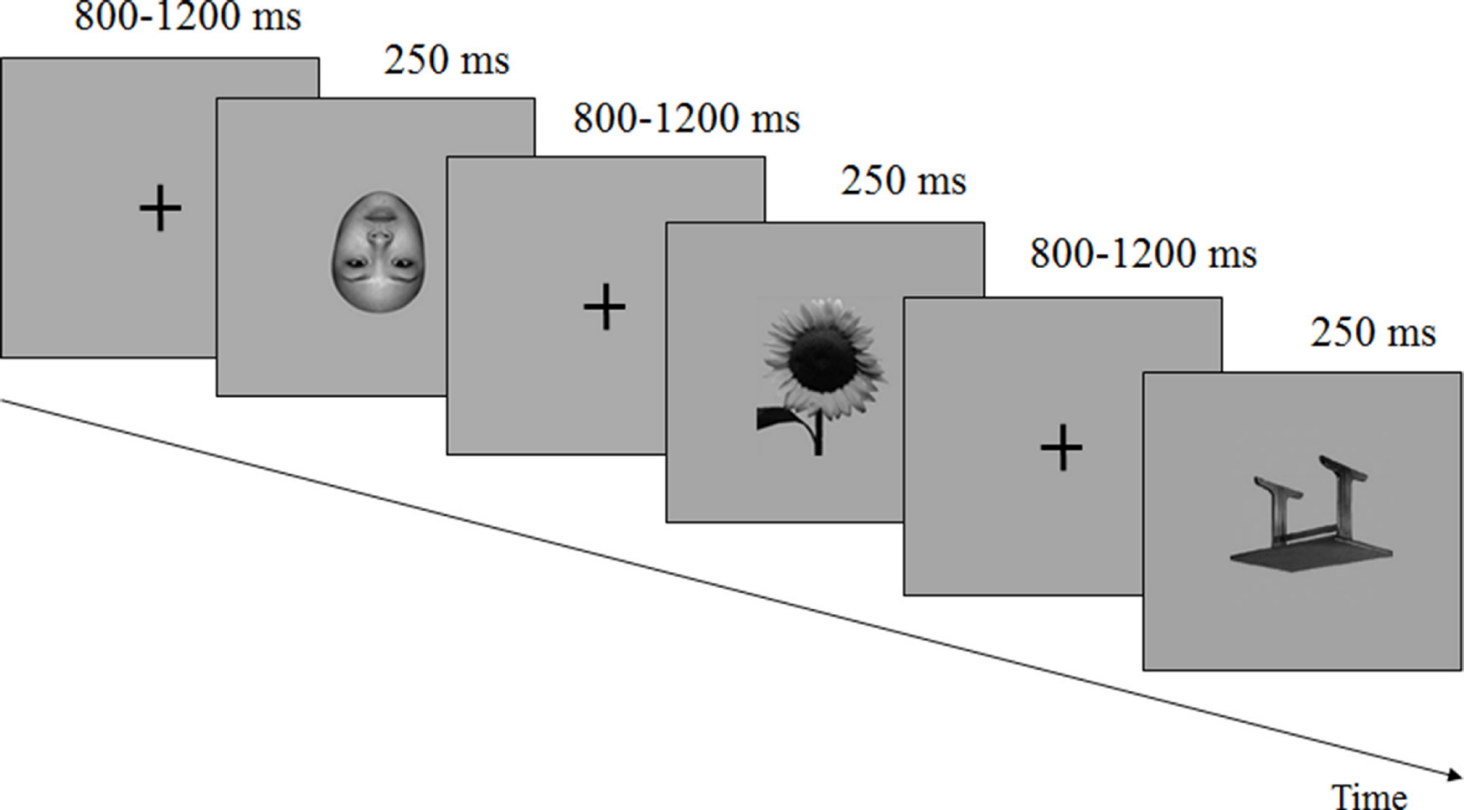
Stimuli Examples of faces and objects, as well as the target flowers used as stimuli.

The stimuli used in the study were 75 photographs of unfamiliar faces, 75 photographs of tables, and 45 photographs of flowers, all in gray-scale. Fifty percent of the faces were male, and 50% were female; all were presented without hair, eyeglasses, or other accessories (Figure 4). Five stimulus conditions were upright faces, inverted faces, upright tables, inverted tables, and upright flowers. All the images were equated for luminance and root-mean-square (RMS) contract with the Photoshop software system (Adobe Systems, Inc., San Jose, CA). The stimuli were presented at the center of a computer screen and viewed from a distance of 1.2 meters at a visual angle of 5.05 degrees vertically and 6.06 degrees horizontally. The study procedure consisted of the presentation of three blocks of 25 faces and 25 tables each, with 15 flowers as the target stimuli for block 1, 14 flowers for block 2, and 16 flowers for block 3, which was counterbalanced within the subjects. All stimuli were pseudo-randomized, and each stimulus was presented for 250 ms, with an inter-stimulus interval randomized to range from 800 ms to 1,200 ms. The subjects were instructed to keep a mental count of the number of flowers in each presentation block. The subjects reported the number of flowers counted at the completion of viewing each block.

### EEG recording

EEG signals were continuously recorded with a DC band pass of 200 Hz and a sampling rate of 1000 Hz using the Cognitrace amplifier (www.ant-neuro.com). An electrode cap with 16 Ag/AgCl electrodes were mounted according to the extended international 10–20 system (F3, Fz, F4, C3, Cz, C4, P7, P3, Pz, P4, P8, PO7, PO8, O1, Oz, and O2) and referenced to the tip of the nose. Vertical and horizontal EOG signals were recorded with two pairs of electrodes, one placed above and below the right eye and the other placed 10 mm from the lateral canthi. Electrode impedance was maintained at less than 5 KΩthroughout the recording phase of the study. The array of electrodes was used to focus on the interest areas that have significant connectivity with the occipito-temporal area, known as the “core system of face perception” [24].

### Data analysis

#### Interregional phase synchronization

Data from each epoch were filtered into alpha (8–14 Hz), beta (15–30 Hz), and low-gamma (30 to 45 Hz) frequency ranges. Alpha, beta and low-gamma band network synchronizations were investigated, as these rhythms are particularly relevant for interregional communication [39–42]. The Hilbert transform operator was employed to obtain the time series of instantaneous phase measures for each trial, sensor and frequency band. Epochs were extracted from 300 ms prior to stimulus onset through 600 ms after stimulus onset. Due to distortions involved in calculating the Hilbert transform at the edges of the analyzed epochs, we did not display the first or last 150 ms (150 sample points) time windows in the synchrony analysis of our epochs. Inter-regional phase-locking was calculated for each sensor pair and frequency using the PLI, which measures the reliability of phase relations between two regions at a given time point relative to stimulus onset. For each electrode pair, i and j, and time point, t (ms), and for all the trials (*n* = 1,…*N*), the connectivity over the trials was indexed by calculating the PLI across trials for each frequency band and subject using the following formula:

$${\mathrm{PLI}}_{\mathrm{ij}}\left(t\right)=\frac{1}{N}|{å}_{n}^{N}=1\mathrm{sign}\left[{\mathrm{Dj}}_{\mathrm{ij}}\left(t,n\right)\right]|$$ (1)

where ${\mathrm{Dj}}_{\mathrm{ij}}\left(t,n\right)$ s is the phase difference of $j_{i}\left(t,n\right)-j_{j}(t,n).$

Connectivity over time is more sensitive for resting-state data or for tasks that have long event durations, such as at least several hundred milliseconds, due to poor temporal precision of connectivity over time. Connectivity across trials has a higher temporal precision and is therefore able to better identify the time course of changes in connectivity and transient changes in connectivity.

This method produces a sensor-by-sensor adjacency matrix for each time point within each analyzed frequency band. Time series of adjacency matrices were compared across groups for each trial condition. These adjacent matrices were used to investigate event-related connectivity dynamics and to identify windows for further statistical analysis.

To visualize our results, we defined the unbiased functional connection in the network with surrogate data. Twenty surrogate data sets were computed in shuffled trials [13, 35]. For a connection pair of i and j, if the distribution of 20 PLI values of surrogate data were deviated from the PLI of original data, the i and j pair was deemed to be a true connection. Otherwise, it was disconnected (*PLI*_*ij*_ = 0). Nonparametric Wilcoxon signed-rank tests were performed such that the median of 20 PLI values of surrogate data were compared with the PLI of original data. (H0 [null hypothesis]: 20 PLI values of surrogate data [${PLI}_{ij}^{surrogate}$] have symmetric distribution with the median µ, where µ is the PLI of original data [${PLI}_{\mathrm{ij}}^{orignal}$]) [43].

$${PLI}_{ij}={PLI}_{ij}^{orignal}-median\left({PLI}_{ij}^{surrogate}\right)\mathrm{if}\;P<0.05{PLI}_{ij}otherwise$$ (2)

### Topographical analysis of phase synchrony

To characterize the event-related network connectivity dynamics, adjacent matrices were averaged for non-overlapping 150 ms time-windows (0–150 ms, 150–300 ms), which represented the mean connectivity within these active windows for each subject. Adjacent matrices were then averaged across an equivalent number of time points (–150 to 0 ms) in the pre-stimulus baseline interval.

The NBS is a network-specific approach to control the FWER in the weak sense when performing mass univariate testing on all connections in a network. The component analysis is in NBS for dealing with the multiple comparisons problem during connectome-wide analysis. The NBS seeks to exploit the topological characteristics of an effect to boost the power with which that effect can be detected. Any such effects on brain networks are likely to encompass multiple connections and nodes, which form interconnected subnetworks. A corollary of this observation is that interesting variations in network connectivity are more likely to span multiple edges rather than to be confined to individual connections. Therefore, we focused on the widespread changes in brain activity rather than the localize effects of individual edges. Traditional methods, such as the Bonferroni or false discovery rate corrections, are overly conservative because they treat each test independently. The NBS evaluates the null hypothesis at the level of connected edge components showing a common effect. This method provides a considerable gain in statistical power. Instead of identifying clusters of voxels in physical space, the NBS identifies connected subnetworks in topological space. The size of a subnetwork is most typically measured by the number of edges that it comprises. The NBS defines these interconnected subnetworks as connected components and ascribes a familywise error corrected *p*-value to each subnetwork using permutation testing [37, 38].

**Figure 5:**
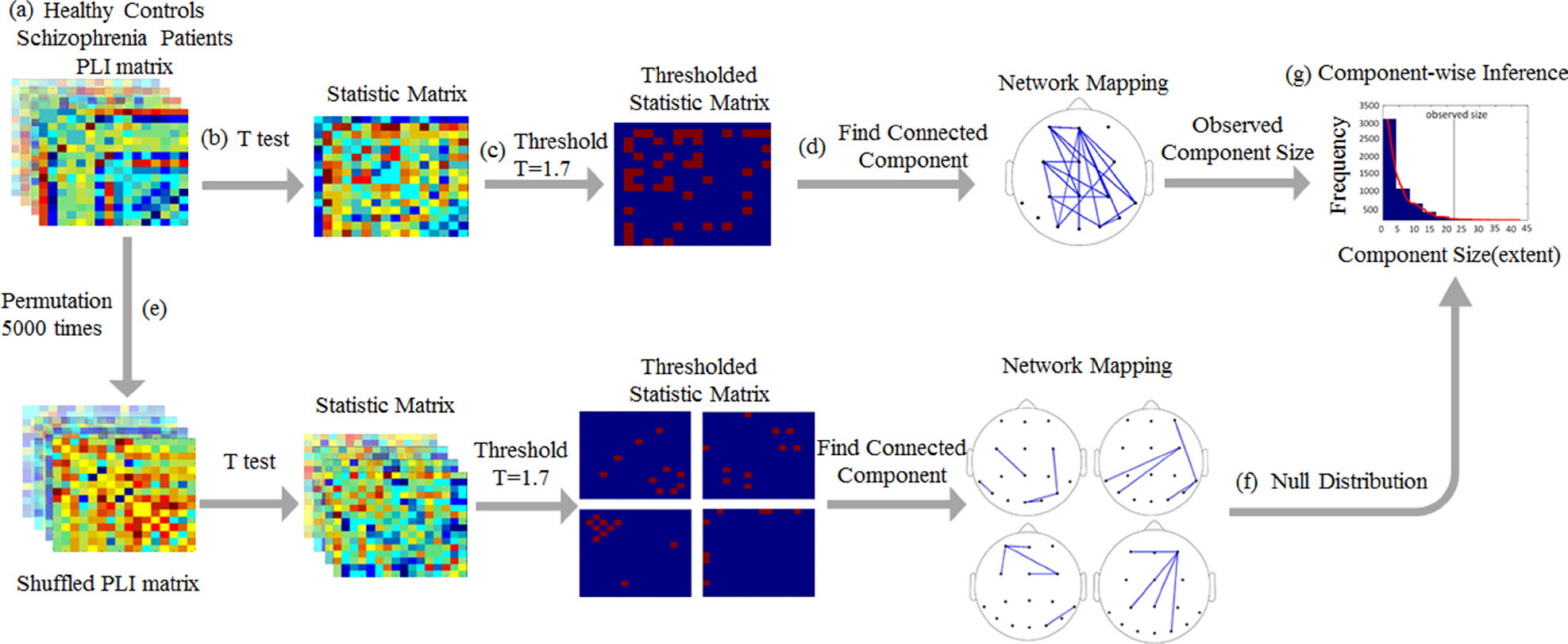
Schematic of the network-based statistics analysis (**A**) Schematic of the comparison of functional connectivity networks in healthy participants and schizophrenia patients, as calculated by the PLI, in the upright face condition. (**B**) A *t*-test statistic was computed at each network edge, resulting in a matrix of statistical values. (**C**) This matrix was then thresholded using a primary, component-forming threshold (T = 1.7, corresponding to *p* < 0.01) to yield a thresholded and binarized statistical matrix. (**D**) The connected components of this thresholded statistical matrix were identified, and the size of each was computed; this component comprises 22 edges. (**E**) The control and patient subjects of were shuffled randomly 5000 times, and steps (b-d) were repeated. (**F**) At each iteration, the size of the largest component was stored to generate an empirical null distribution of maximal component sizes. (**G**) The observed size of the component illustrated in (d) corresponded to *P =* 0.0150. The functional connectivity differences between the patients and the controls involve a distributed network of connections (d) for the frontal, central parietal, temporal and occipital areas.

The detailed step of the NBS is shown in Figure 5. In the present study, the initial univariate threshold for between-group comparisons was adapted for the data distributions being analyzed to T = 1.7, corresponding to a *p*-value of *p <* 0.01, while effective control for multiple comparisons was achieved irrespective of this initial threshold. Data surrogation was repeated 5,000 times to create a null distribution. In this case, to explore the temporal evolution of connectivity changes between the groups, we subtracted the mean baseline adjacency matrix from the mean active window adjacency matrix for each subject.

### Graph theoretical analysis of dynamic network topologies

Functional connectivity among sensors was measured by computing the PLI for every possible pair during the active and baseline time windows. The resulting nonlinear correlation matrices were converted to weighted graphs.

To characterize the event-related weighted network dynamics, we constructed an active and baseline network G (16 × 16) for each subject using GRETNA (http://www.nitrc.org/projects/gretna/) [44]. In the present study, global threshold methods (0.3 ≤ S ≤ 0.40) were used to distinguish real connections from spurious connections, which altered the network connection density. The clustering coefficient is essentially the ratio of the geometric weight of all closed triplets to the total weight of both open and closed triplets in the network [18, 45]. The clustering coefficient was calculated with the following formula:

Ci=åiÎGåi,hÎG(WijWihWjh)13ki(ki−1)/2 (3)

where *k*_*i*_ is the degree of node I, and *W*_*ij*_ is the weight difference between node i and node j in the network. The mean clustering coefficient of network C is the average over each node’s clustering coefficient, reflecting the level of local connectedness of a node.

$$C=\frac{1}{N}C_{i}$$ (4)

The clustering coefficient for a given network was obtained by averaging all clustering coefficients of individual nodes.

To address the dependence of network measures on the connectivity density, we normalized the clustering coefficients with respect to 100 randomized networks matched to the empirical network for size, connection density, and degree distribution.

We integrated the normalized clustering coefficient across the full range of thresholds analyzed to yield the area under the curve (AUC) rather than on each of the many threshold-specific measures to avoid the multiple comparisons problem. Statistical testing was then performed on the event-related AUC of the normalized clustering coefficient, calculated by subtracting the baseline AUC from the active AUC, indicating changes from a baseline period.

### Statistical analysis

SPSS version 20.0 (SPSS, Inc, Chicago, IL) was used for statistical analysis (http://www.spss.com/). Repeated-measures ANOVA was carried out separately for the event-related AUC of the clustering coefficient, path length and small-worldness involving Group (schizophrenia patients vs. healthy controls) as the between-subjects factor and Category (faces vs. tables) and Orientation (upright vs. inverted) as the within-subject factors. The Greenhouse-Geisser epsilon value in which the repeated-measures data failed the sphericity test was obtained in all cases [46]. All statistical comparisons were two-tailed with α = 0.05. We used the Bonferroni correction to correct for the effect of multiple comparisons in neural oscillations.

## Abbreviations

(PLI): phase lag index
(fMRI): functional magnetic resonance imaging
(EEG): electroencephalography
(DTI): diffusion tensor tractography
(NBS): network-based statistic
(PET): positron emission tomography
(DSM-IV): Diagnostic and Statistical Manual of Mental Disorders, Fourth Edition
(PANSS): Positive and Negative Syndrome Scale
(PSP): Personal and Social Performance scale
(RMS): root-mean-square
